# Fusion localization for indoor airplane inspection using visual inertial odometry and ultrasonic RTLS

**DOI:** 10.1038/s41598-023-43425-y

**Published:** 2023-10-23

**Authors:** Ingyoon Park, Sangook Cho

**Affiliations:** Koreanair R&D Center, Daejeon, South Korea

**Keywords:** Aerospace engineering, Electrical and electronic engineering

## Abstract

In this paper, the fusion localization system for the visual inertial odometry (VIO) and ultrasonic real-time localization system (RTLS) for indoor airplane inspection using drones is proposed. In a hangar environment, either trilateration-based RTLS or vision-based localization shows disadvantages and neither can be used alone. In this research, we design a configuration of VIO suitable for hangar environment and outlier filter on ultrasonic RTLS for non-line of sight situations, so that both can be fused using graph optimization. The proposed solution can provide more accurate localization than the visual odometry-only system as well as continue estimating positions in the absence of RTLS data. Localization and real-time performance of the proposed algorithm are evaluated through experimentation in a hangar and a flight test in an outdoor space.

## Introduction

Currently, there is substantial research on and application of utilizing drones for visually inspecting construction sites, bridges, factories, etc. Using drones to inspect airplanes is attracting much attention as it automates inspections, increasing maintenance timeliness and worker safety. Airplane inspections are commonly conducted in hangars where Global Positioning Systems (GPS) are not available. To address this, indoor localization solutions are needed for automated airplane inspection which can be conducted by drones.

The Indoor real-time localization system (RTLS) can be employed as a localization solution in a hangar. One type of indoor RTLS is Received Signal Strength Indicator (RSSI) technology using Wi-Fi or Bluetooth signal. The RSSI measures the distance between a transmitter and a receiver using the strength of the received signal, but it is susceptible to significant errors, especially in areas with obstacles or radio frequency (RF) signal interference. Another approach is ultrasonic or UWB (ultra-wideband) based RTLS which measures the signal’s time of flight (ToF). This offers more accurate and stable performance compared to RSSI-based solutions. Particularly, UWB employs impulse radio frequency carrier-less signals, allowing it to measure time between transmit and receive with high accuracy^1^. However, UWB or ultrasonic RTLS can also be affected by signal degradation due to obstacles in the hangar, multipath effect near airplane surfaces, or signal interference from avionic equipment in an airport^1,2^.

Odometry or SLAM (Simultaneous Localization and Mapping) based localization using LiDAR (Light Detecting and Ranging) or a camera onboard a drone can be a solution for indoor drone inspection. However, these approaches also suffer from drift errors due to the lack of feature points on airplanes and the large space of a hangar. Regarding the design of an onboard localization system for an airplane inspection drone, Size, weight, and Power (SWaP) is an important factor to consider. Drones with low SWaP are suitable for composing a multi-drones system which can accelerate airplane inspection speed and efficiency. Onboard localization system using three dimensions LIDAR has high SWaP due to the heavy weight of LIDAR sensors and the computing load to process point cloud data^3^. On the other hand, a vision-based onboard camera localization system has the advantage of having a smaller SWaP compared to one with a LIDAR system.

To achieve indoor localization in a hangar, various approaches from related studies can be considered. One such approach is a combined laser and vision-aided inertial navigation system^4^. In this combined system, laser SLAM and visual SLAM are integrated, but they can accumulate errors when loop closures do not occur. Considering the low chances of revisiting the same place in the inspection flight path, another error compensation method rather than loop closure is needed. Another approach is indoor localization using fiducial markers attached to warehouse walls, as proposed in^5^. However, in a hangar, fiducial markers installed on the walls may be too small to provide accurate reference positions due to the hangar’s large space, resulting in excessive noise. As for RTLS usage, Wi-Fi trilateration for warehouse localization is evaluated and tested in^6^. It is important to note that the author mentions that Wi-Fi signal attenuation and reflections affect the accuracy. Therefore, we aim to propose a fusion localization system that combines drone onboard odometry with ultrasonic or UWB-based RTLS to compensate for error accumulation.

As for research on localization in a hangar for airplane inspection, Pugliese et al.^7^ proposed LIDAR-based localization taking advantage of geometric knowledge of the target airplane’s fuselage. However, the algorithm used in this proposed methodology cannot produce three-dimensional Euclidean coordinates; it only provides the distance information from an airplane fuselage cylinder axis to a drone. Tappe et al.^8^ implemented an indoor hangar localization by LIDAR-based LoLa SLAM (Low-Latency SLAM)^9^. However, the authors also commented that further research on fusing with another sensor could be helpful as a laser has drawbacks in measuring range on some materials such as glass or mirrors.

In this paper, we propose the fusion of an ultrasonic trilateration-based RTLS with the visual inertial odometry (VIO) within a hanger. We configure the VIO system for hangar use, including camera type, camera direction, and visual odometry (VO) algorithm. To handle non-line-of-sight (non-LOS) situations, we design an RTLS outlier filter that uses VIO as a reference. Localization from VIO and outlier-filtered RTLS is fused using a graph optimization algorithm. The contributions of this research are as follows:

- Accumulated drift error of VIO is compensated by RTLS
- In the absence of RTLS data, localization can continue by VIO
- VIO configuration suitable for a hangar is proposed
- An outlier filter to reject abnormal values from RTLS is designed
- Real-time performance is achieved and tested through a flight test

**Figure 1:**
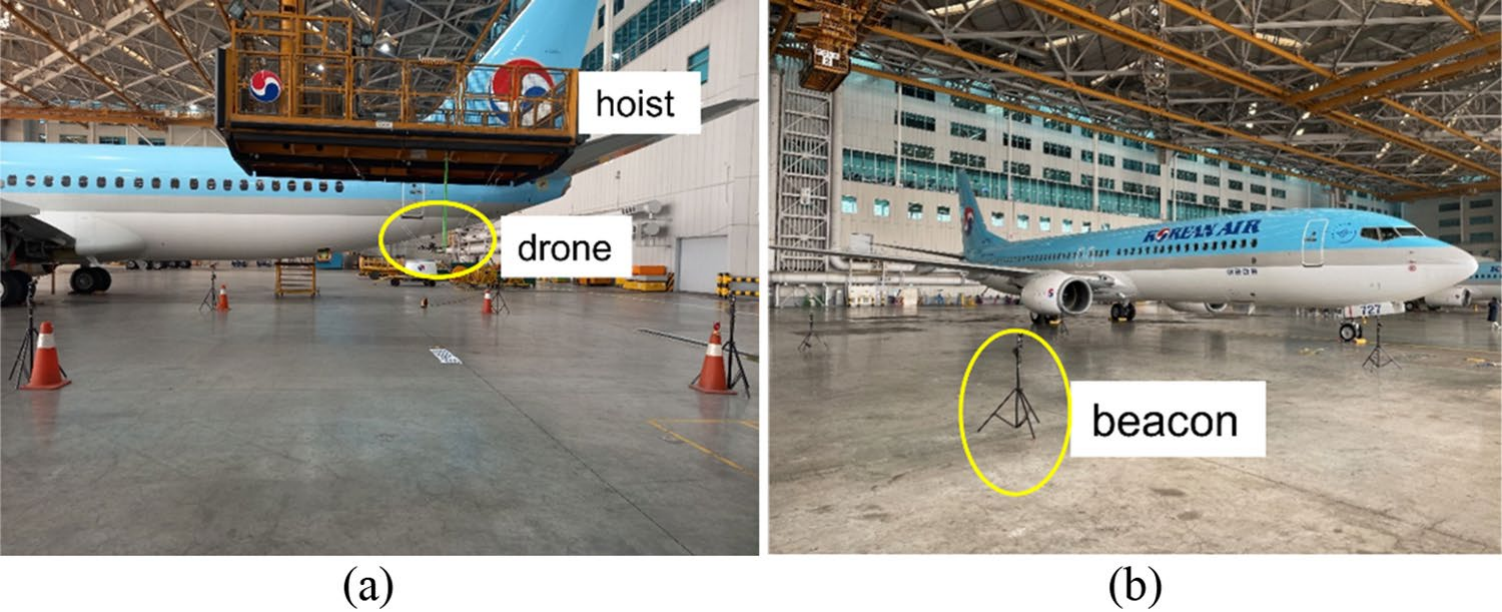
Test environment in a hangar.

The organization of this paper is as follows: "Localization system design for an airplane inspection drone" section proposes a fusion localization system that combines VIO and ultrasonic RTLS for airplane inspection drones. "Experiment" section describes the hangar tests (Fig. 1) and the outdoor flight tests for the suggested fusion localization system. "Result" section shows that the suggested solution can continue localization even when ultrasonic RTLS positions are abnormal. "Result" section also shows that fusion with the ultrasonic RTLS has greater accuracy than VIO alone, and its capability to perform a real-time flight.

## Localization system design for an airplane inspection drone

### Fusion system architecture

Ultrasonic RTLS systems offer precision within a few centimeters, which is sufficient for indoor drone navigation^10^. However, when drones fly close to airplanes, ultrasonic waves can be disrupted or reflected by an airplane's fuselage or wings, resulting in unstable RTLS localization. This necessitates the use of secondary localization.

As for VO-based localization, their pose output period is faster than ultrasonic RTLS and the calculation results are more precise in a short time duration. In a hangar environment, however, VO suffers from a lack of feature points due to the uniform color painting on the airplane’s surfaces and the insufficient presence of objects such as maintenance tools and equipment on a hangar floor. In addition, VO needs to eliminate the drift error, which accumulates gradually, using a compensation algorithm such as loop closure, which occurs when the same place is visited again^3^. However, loop closure may not occur in an automated inspection flight path, so another method is necessary to compensate for VO’s error in airplane inspection applications.

**Figure 2:**
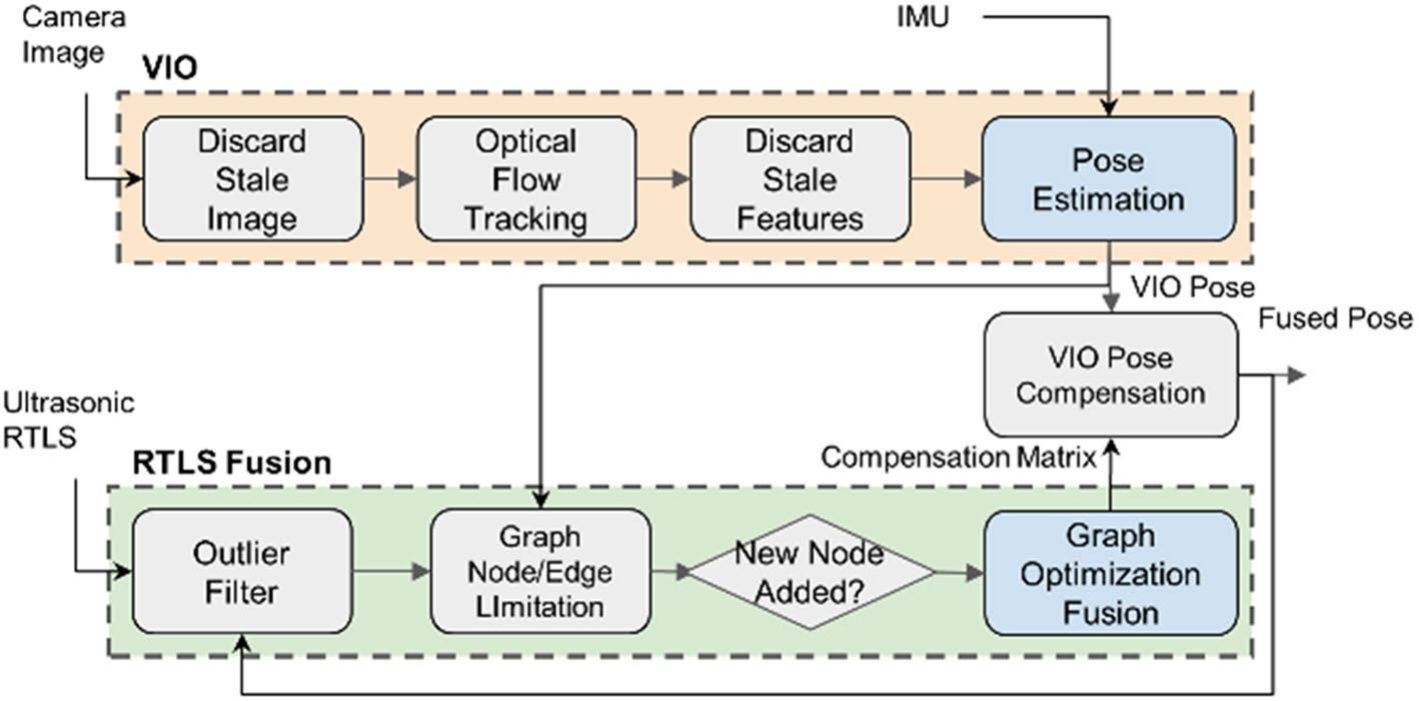
Fusion algorithm.

Therefore, to take advantage of both RTLS and visual odometry, we propose a fusion localization system that combines ultrasonic RTLS and stereo camera VIO, using an outlier filter and graph-based optimization as described in Fig. 2. Regarding VIO, we implement buffer management on images and features to ensure real-time capability. We opt for an optical flow-based VIO algorithm due to its high localization accuracy in the context of airplane inspection missions. Detailed VIO configuration adequate for airplane inspection application in a hangar is described in "VIO configuration" section. As for ultrasonic RTLS, we address unstable position data in the case of non-LOS and ultrasonic reflection through the outlier filter which is described in "Ultrasonic RTLS" section. Graph optimization design is explained in "Graph optimization fusion" section. When formulating a graph consisting of RTLS and VIO, we apply size marginalization by limiting the number of graph nodes and edges (Fig. 2) to maintain real-time computational capabilities.

### VIO configuration

VIO configuration for localization in a hangar environment is analyzed and selected with respect to camera direction, camera type, and visual odometry algorithm. An inspection drone equipped with a VIO localization system has two types of cameras: a VIO camera and an inspection camera. The VIO camera uses a global shutter image sensor to capture moving objects without distortion, while the inspection camera uses a rolling shutter image sensor to capture high-resolution still images. Because this research focuses on a VIO configuration, the term "camera" refers to the VIO camera.

#### Camera direction

**Figure 3:**
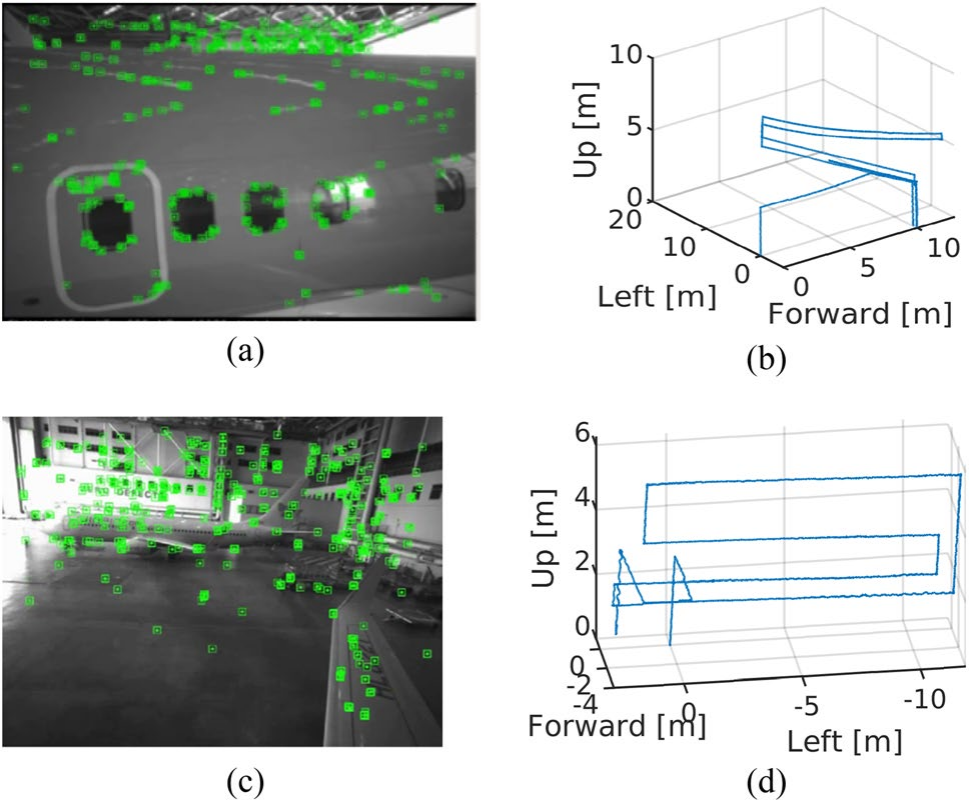
Visual odometry and result trajectory of Aircraft view (**a**,**b**) and hangar view (**c**,**d**) by ORB-SLAM2^11^ algorithm without loop closure.

Regarding camera direction, a camera direction configuration turning away from the airplane yields the best VO performance. In airplane inspection missions, inspection drones typically fly at a distance of 2–3 m from an airplane’s body. As a result, if a camera is directed toward the airplane, the field of view (FOV) of a camera is blocked by the airplane’s body, causing a lack of feature points for VO calculation. In addition, lights from the hangar ceiling can be reflected by the surface of an airplane body, resulting in distortions in the vision-based pose estimation. The images in Fig. 3a and c show detected feature points, while Fig. 3b and d display the resulting trajectories obtained using stereo images when a drone hanging from a hoist moves closely around a target airplane as in Fig. 1a. It can be seen in Fig. 3a that feature points are sparse on the airplane fuselage and light is reflected on the upper body of an airplane when a direction configuration is toward an airplane. At around 5 m in height, the light reflection on the upper body of the airplane causes the estimated trajectory to become distorted with a curved line when it should be straight (Fig. 3b).

On the other hand, when the camera is turned away from the airplane, thanks to the clear FOV, more feature points are visible on hangar walls, other aircraft, and maintenance objects on the hangar floor (Fig. 3c). As seen in Fig. 3d, the trajectory is well estimated due to the additional feature points and exhibits no curved lines. It is important to note that if there are only a few objects on a floor within the view of the camera, VO may have to rely solely on hangar wall features, which could lead to a pose estimation inaccuracy. In such cases, fusion with RTLS for compensation of VO drift is crucial.

The last direction configuration that can be considered is toward the ceiling of the hangar. Although the ceiling has plenty of feature points on the beam structures as can be seen in Fig. 3a, high ceiling heights (e.g., 20 m) are prone to scale error in VO calculation. Thus, using the ceiling view for camera direction configuration may not be appropriate, leading to our decision to use a camera direction turned away from the airplane.

#### Camera type

Comparing Red Green Blue-Depth (RGB-D), mono, and stereo cameras for VO, the stereo camera is the most suitable camera type for airplane drone inspection because stereo VO can utilize the view turned away from an airplane and recover the scale in translation calculation.

**Figure 4:**
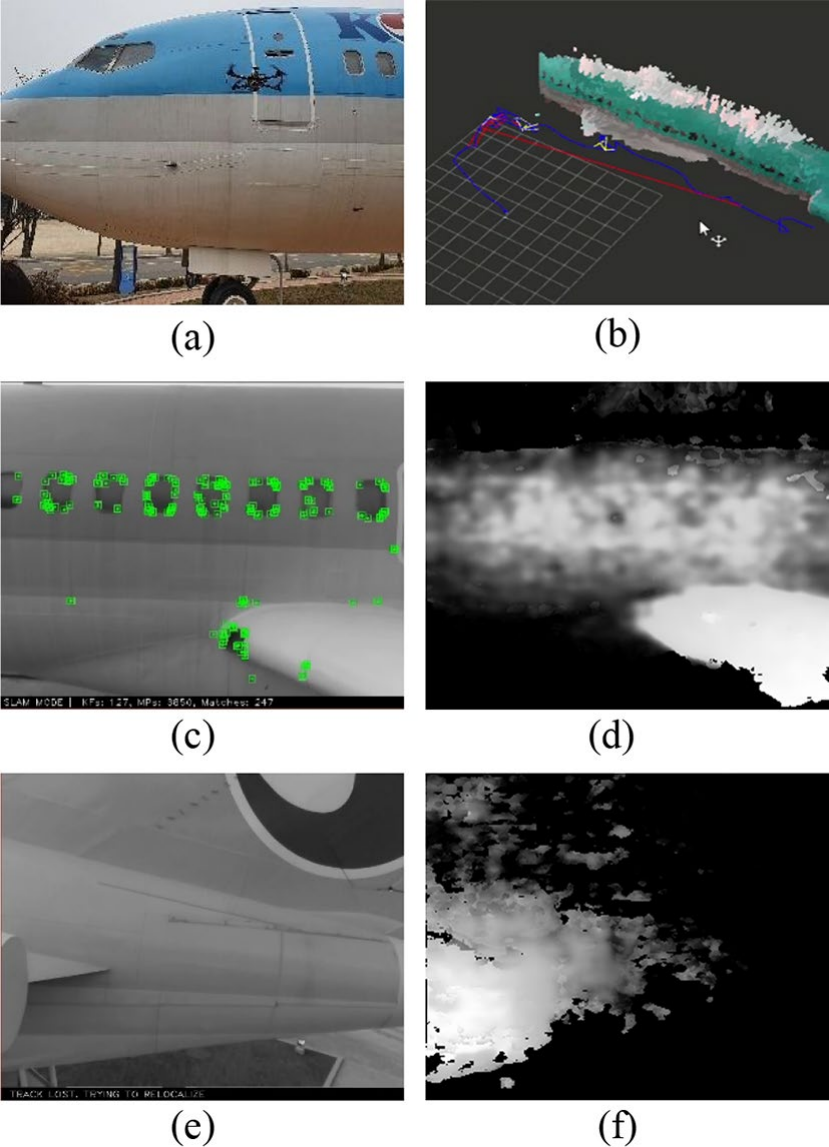
RGB-D visual odometry experiment: manual test flight (**a**), 3D reconstruction by RTAB-MAP^12^ algorithm (**b**), feature tracking on the fuselage by ORB-SLAM2 algorithm (**c**), RGB-D data on the fuselage (**d**), tracking lost on airplane tail by ORB-SLAM2 algorithm (**e**), and RGB-D data on tail (**f**).

Although a RGB-D camera has the advantage of providing depth information, it has a limited effective range. Due to the range constraints, the camera view should be directed toward the aircraft, leading to feature point deficiencies in VO. For evaluation of RGB-D-based VO, a drone flight was conducted while fixing its camera view to a Boeing 727-200S airplane installed outdoors. RGB-D data were acquired by onboard Intel Realsense D435i which has an effective range limit of around 5 m (Fig. 4a,b). The results of the RGB-D VO shows that it can continue to track features located on windows in the fuselage part despite not having enough features (Fig. 4c,d). However, in the tail part where there are no windows, VO loses its tracking due to a lack of feature points as shown in Fig. 4e and f.

Mono and stereo cameras can both be considered for VO when capturing images while facing away from an airplane. Stereo cameras are more suitable for VO due to their capability to recover scale in translation, which mono cameras lack. Evaluation of various Stereo VO algorithms is detailed in “Visual odometry algorithm” section.

#### Visual odometry algorithm

To select the most suitable visual localization algorithm for airplane inspection missions, we compared the performance of feature-based stereo VO and VIO algorithms and optical flow-based stereo VO and VIO algorithms. As for a feature-based visual localization algorithm, ORB-SLAM2 (VO) using stereo images and ORB-SLAM3 (VIO)^13^ using stereo images and inertial measurement unit (IMU) data are tested. As for an optical flow-based visual localization algorithm, VINS-Fusion^14^ VO and VINS-Fusion VIO are evaluated.

In the test environment of Fig. 1b, a drone was moved by walking at a constant speed along the reference trajectory A shown in Fig. 6a. The reference trajectory followed lines on the floor of the hangar, and its length was measured using a laser ranger. It had a rectangular shape with dimensions of 16.2 by 7.2 m. The total distance covered by the drone during the test was 46.8 m. During the test, stereo images were acquired using Intel Realsense D435i, and inertial data were collected from the IMU sensor in Pixhawk FCC (flight control computer). The trajectory estimations of the candidate VO and VIO algorithms were performed from the data. The corner points from both the rectangular reference positions and the estimated positions were matched and synchronized. The positions on the side of the rectangular trajectory were sampled using linear interpolation because the drone was moved at a constant speed. Root mean square error (RMSE) on horizontal coordinates was calculated for each candidate VO and VIO algorithm using the sampled reference and estimated positions. The RMSE results were then compared to determine the relatively best algorithm, rather than an absolute evaluation.

**Figure 5:**
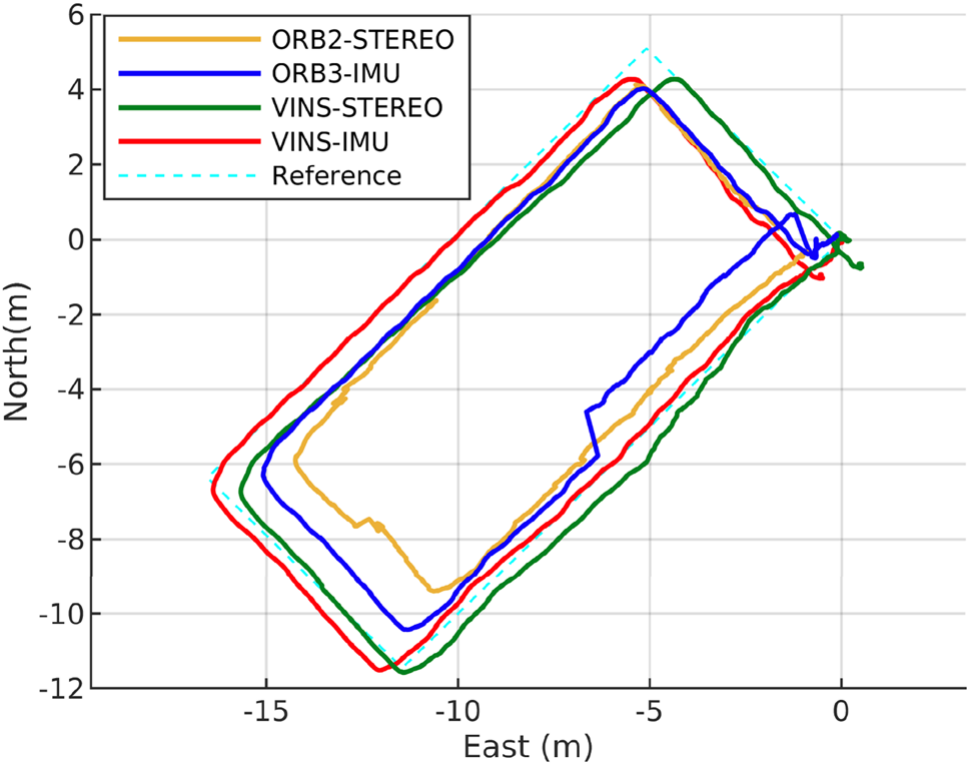
VIO algorithm trajectory result comparison.

Figure 5 and Table 1 show that among candidate algorithms, VINS-Fusion VIO, which is an optical flow-based algorithm, exhibits the smallest RMSE. The reason for VINS-Fusion’s high performance is that it uses a sparse optical flow-based Lucas-Kanade algorithm^15^, which is suitable for slow movement during airplane inspection flights. Small position deviations by slow movement are crucial in optical flow because they satisfy Taylor series approximation in optical flow algorithms and can help to avoid the aliasing problem associated with sparse optical flow^16^.

**Table 1:** VO algorithm performance comparison.

	VINS-Stereo	VINS-Stereo IMU	ORB-SLAM2 Stereo	ORB-SLAM3 Stereo IMU
RMSE (m)	0.7402	**0.5995**	1.3729	1.0840

Significant values are in bold.

Optical flow estimates the motion vector of points using the brightness constancy condition of pixels within consecutive images. Considering pixel brightness intensity $I\left(x,y,t\right)$ which is located in $x,y$ in an image at time $t$, brightness constancy condition is expressed as

1 $$I\left(x,y,t\right)= I\left(x+dx,y+dy,t+dt\right)$$

Assuming motion between consecutive images is small, Taylor series approximation can be applied,

2 $$I\left(x+dx,y+dy,t+dt\right)= I\left(x, y,t\right)+\frac{\partial I}{\partial x}dx+\frac{\partial I}{\partial y}dy+\frac{\partial I}{\partial t}dt$$

Then, optical flow can be derived as below

3 $${I}_{x}u+{I}_{y}v+{I}_{t}=0, where \; \; \,u=\frac{dx}{dt}, v=\frac{dy}{dt}, {I}_{x}=\frac{\partial I}{\partial x},{I}_{y}=\frac{\partial I}{\partial y}, {I}_{t}=\frac{\partial I}{\partial t}$$

$u, v$ mean the optical flow of $I\left(x,y,t\right)$, and ${I}_{x},{I}_{y}, {I}_{t}$ denote spatial and time derivatives of $I\left(x,y,t\right)$.

In conclusion, we choose an optical flow-based VIO algorithm based on its superior performance in inspection flights. This choice was made as optical flow-based VO outperforms feature-based VO, and VIO offers greater accuracy than VO due to its tight coupling with the IMU.

#### Real-time implementation

To achieve real-time performance in VIO, marginalization on images and features is implemented as shown in Fig. 2, and GPU resources are utilized for feature tracking. Since bundle adjustment in VIO demands substantial computing power, the target board for this research, the Jetson Xavier NX, occasionally struggles to process all the features arriving at 15 Hz. If the delayed images and feature data pile up, the time offset between real motion and VIO output increases. Therefore, an image and feature buffer marginalization logic is implemented to prevent a delay between real motion and VIO output by discarding old images and features when new ones arrive before old ones have been processed. In addition, to alleviate the high processing load on the CPU which has limited resources, and improve overall speed, we employ GPU acceleration in the feature tracking process^17^.

### Ultrasonic RTLS

**Figure 6:**
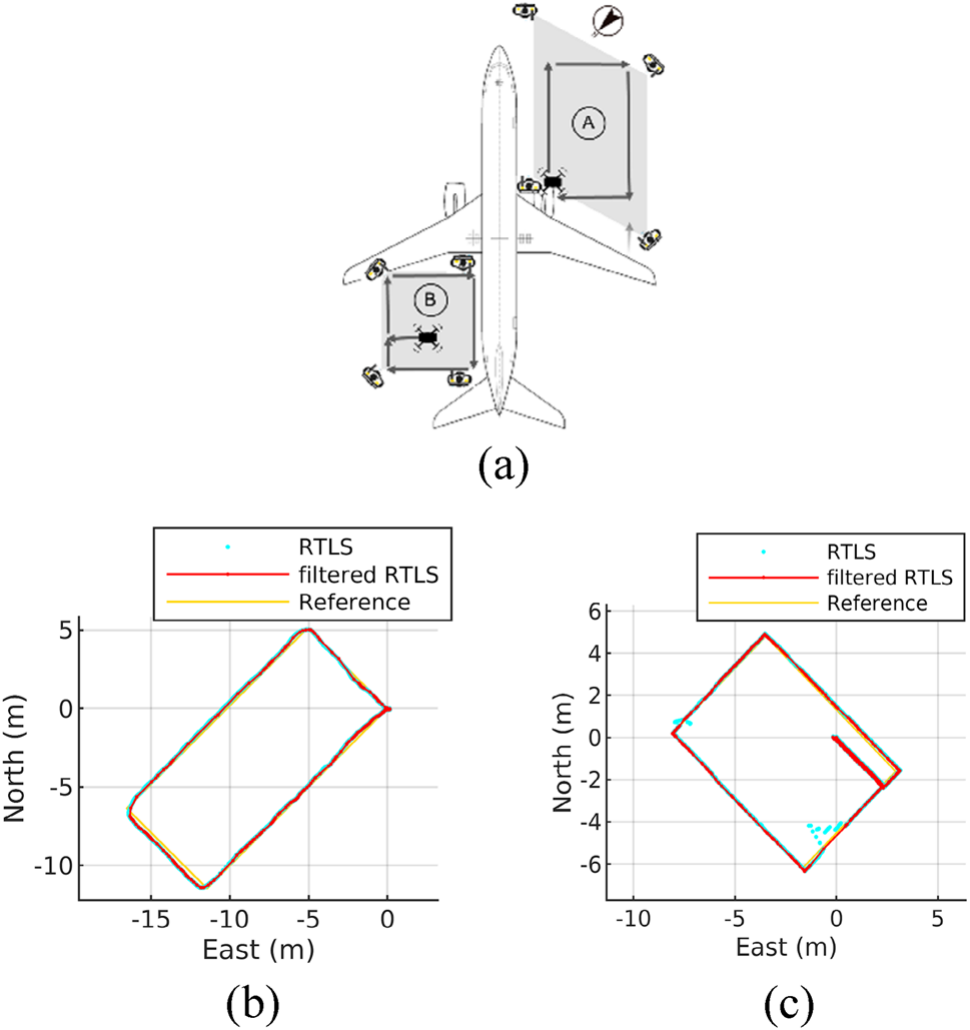
Ultrasonic RTLS test configuration in a hangar (**a**) and its position output (normal output (**b**) and irregular output (**c**)).

Ultrasonic beacon indoor GPS from Marvelmind^10^ is used in a hangar RTLS owing to its small and light SWaP. As in Fig. 6a, a system suite for each flight area comprises one rover attached to a drone and four stations installed on the hangar floor. Because no beacon stations for vertical position measurement are installed in the test, only planar X and Y positions from ultrasonic RTLS localization are utilized, while vertical Z positions are not used.

In the trajectory A test (Fig. 6b), which was conducted without non-LOS conditions as shown in Fig. 1b, RTLS provides normal localization values. However, in the trajectory B test (Fig. 6c), RTLS localization is momentarily unstable due to non-LOS, caused by the crane and the drone installation setup on the crane as shown in Fig. 1a.

To address this issue, we’ve implemented an outlier filter (Table 2) that utilizes VIO position output as a reference. This filter rejects abnormal position deviations and repeated same position data (Fig. 6c). The outlier filter takes the inputs of ${{\varvec{p}}}_{cur}^{rtls}, {{\varvec{p}}}_{cur}^{vio}, {{\varvec{p}}}_{prv}^{w},\Delta {t}_{rtls},\Delta {t}_{vio}$ which denote the current RTLS position output, current VIO position output, previous fusion estimated world position output, RTLS output time interval, and VIO output time interval, respectively. This filter outputs $rtls\_good$, which indicates the validity of the RTLS position output, in order to discard RTLS output when $rtl{s}_{good}=0$. In this filter, considering the asynchronous arrival time of RLTS and VIO values, they are compared in the form of velocity, denoted as ${{\varvec{v}}}_{rtls},\boldsymbol{ }{{\varvec{v}}}_{vio}$, where ${{\varvec{v}}}_{rtls}$ represents the position difference of previous fusion estimated state and RTLS, and ${{\varvec{v}}}_{vio}$ represents the position difference of previous fusion estimated state and VIO, respectively. Because VIO can provide an accurate position in a short amount of time, we reject the RTLS measurements if the difference between the ${{\varvec{v}}}_{rtls}$ and the ${{\varvec{v}}}_{vio}$ is greater than the threshold as described in the Eq. (4). The tolerable difference limit of RTLS and VIO is the sum of the RTLS error part (${v}_{rtls\_offset}$), related to its precision, and VIO error part (${v}_{vio\_tol}$) associated with its drift accumulation characteristic. These values are recalculated at every filter execution time.

**Table 2:** Ultrasonic RTLS outlier filter.

Algorithm 1: Ultrasonic RTLS outlier rejecter	
	***Input***: ${{\varvec{p}}}_{cur}^{rtls}, {{\varvec{p}}}_{cur}^{vio}, {{\varvec{p}}}_{prv}^{w},\Delta {t}_{rtls},\Delta {t}_{vio}$
	***Output:*** $rtls\_good$
	***Param***: *drift_scale, rtls_precision*
1:	${{\varvec{v}}}_{rtls}\leftarrow \boldsymbol{ }({{\varvec{p}}}_{cur}^{rtls}-{{\varvec{p}}}_{prv}^{w})/$ $\Delta {t}_{rtls}$
2:	${{\varvec{v}}}_{vio}\leftarrow ({{\varvec{p}}}_{cur}^{vio}-{{\varvec{p}}}_{prv}^{w})/\Delta {t}_{vio}$
3:	${\Delta {\varvec{p}}}_{accum}^{vio}\leftarrow \boldsymbol{ }{\Delta {\varvec{p}}}_{accum}^{vio}+{{\varvec{p}}}_{cur}^{vio}-{{\varvec{p}}}_{prv}^{w}$
4:	${v}_{rtls\_offset}\leftarrow \left|rtls\_precision/\Delta {t}_{rtls}\right|$
5:	${v}_{vio\_tol}\leftarrow \Vert {\Delta {\varvec{p}}}_{accum}^{vio}/\Delta {t}_{vio}*drift\_scale\Vert$
6:	***if*** $\Vert {{\varvec{v}}}_{vio}-{{\varvec{v}}}_{rtls}\Vert >{v}_{vio\_tol}+{v}_{rtls\_offset}$
7:	$rtls\_good\leftarrow 0$
9:	***else***
10:	$rtls\_good\leftarrow 1$
11:	${\Delta {\varvec{p}}}_{accum}^{vio}\leftarrow \{0, 0\}$
12:	***end***

4 $$\Vert {{\varvec{v}}}_{vio}-{{\varvec{v}}}_{rtls}\Vert >{v}_{vio\_tol}+{v}_{rtls\_offset}$$

For the ultrasonic RTLS error (${v}_{rtls\_offset}$), we calculate it by dividing the precision of RTLS, which is 2 cm according to the Marvelmind beacon specification^10^, by the time span. VIO error (${v}_{vio\_tol}$) is calculated by multiplying accumulated VIO velocity by a parameter ($drift\_scale$), accounting for accumulating characteristics of VIO drift. If no compensation on VIO estimation by RTLS occurs, we use accumulated VIO translation (${\Delta {\varvec{p}}}_{accum}^{vio}$) to calculate VIO error. VIO translation is reset to 0 if compensation occurs. The outlier filter starts running after an initial duration from take-off because VIO is prone to high errors by restricted camera FOV when the drone is closed to the ground.

### Graph optimization fusion

**Figure 7:**
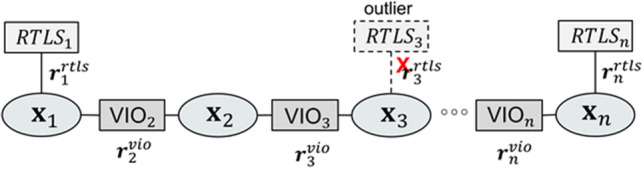
Graph structure.

Graph-based optimization is adopted as the fusion algorithm of VIO and RTLS for localization in a hangar. The graph is composed as shown in Fig. 7 and its nodes and edges are updated with VIO pose estimation and outlier filtered ultrasonic RTLS position. When a new RTLS position is inserted in the graph, an optimization process is conducted. In the graph, ${{r}_{i}}^{vio}$ and ${{r}_{i}}^{rtls}$ means the VIO residual and the RTLS residual at frame $i$, respectively.

If EKF (extended Kalman filter)^18^ is utilized to fuse RTLS and VIO, in the absence of RTLS, the filter continues to execute using VIO only, resulting in high error or unstable output in EKF estimation. On the other hand, the graph optimization method can stop executing optimization when RTLS is unavailable, and restart optimization when RTLS is available again. It utilized the latest graph, including VIO edges that were inserted during RTLS absence.

In this research, to achieve real-time performance in optimization calculation, the total number of nodes is constrained up to 500 using window management, which discards old nodes and edges. Because the period of RTLS localization output (8–9 Hz) is slower than VIO (15 Hz), graph optimization is conducted in the background when new RTLS data updates the graph.

For the graph optimization algorithm, VINS-Fusion graph optimization^14^ is used as the backbone algorithm. State set, which represents the graph nodes, is expressed as $\chi =\{{X}_{0},{X}_{1},{X}_{2},...,{X}_{n}\}$ where $i$-th state ${X}_{i}$ consists of global coordinate position ${{p}_{i}}^{w}$ and orientation ${{q}_{i}}^{w}$. The graph comprising state set $\chi$ and measurement set from RTLS and VIO is interpreted into a non-linear least square problem to estimate the states as below:

5 $${\chi }^{*}=\underset{{\varvec{\chi}}}{\mathrm{argmin}}(\sum_{i\in S1}\parallel {{r}_{i}}^{vio}(\chi ){{\parallel }_{{{\Omega }_{i}}^{vio}}}^{2}+\sum_{i\in S2}\parallel {{r}_{i}}^{rtls}(\chi ){{\parallel }_{{{\Omega }_{i}}^{rtls}}}^{2} )$$

where $\parallel r(\chi ){\parallel }_{\Omega }$ is Mahalanobis norm distance of residual $r(\chi )$, and $S1$ and $S2$ are index sets of VIO and ultrasonic RTLS data, respectively. $\Omega$ is the covariance matrix for VIO or RTLS output. We used a unified diagonal covariance matrix with a value of 0.05 because the Marvelmind system’s RTLS precision is known to be a few decimeters^10^. The VIO covariance matrix is also set to a diagonal matrix with values of 0.1 and 0.01 for position entries and attitude entries, respectively. The residual of VIO is

6 $${{r}_{i}}^{vio}(\chi )=\left[\begin{array}{c}{{q}_{i-1}^{vio}}^{-1}({p}_{i}^{vio}-{p}_{i-1}^{vio})\\ {{q}_{i-1}^{vio}}^{-1}{q}_{i}^{vio}\end{array}\right]\ominus \, \left[\begin{array}{c}{{q}_{i-1}^{w}}^{-1}({p}_{i}^{w}-{p}_{i-1}^{w})\\ {{q}_{i-1}^{w}}^{-1}{q}_{i}^{w}\end{array}\right]$$

where ${p}_{i}^{vio}and$ ${q}_{i}^{vio}$ denote the VIO estimated position and rotation in the VIO local coordinate, while ${p}_{i}^{w}and$ ${q}_{i}^{w}$ denote graph optimized position and rotation in the global coordinate, respectively. $\ominus$ operation calculates pose difference. The VIO position residual takes the position in the displacement form, i.e., ${p}_{i}^{vio}-{p}_{i-1}^{vio}$, rather than position form, because the VIO position accumulates drift. The RTLS residual is

7 $${{r}_{i}}^{rtls}\left(\chi \right)={p}_{i}^{rtls}-{p}_{i}^{w}$$

where ${p}_{i}^{rtls}$ is the RTLS position measurement in global coordinate.

This nonlinear least square problem is solved using Ceres solver^19^ utilizing the Gaussian-Newton and Levenberg-Marquadt^20^ iterative method.

## Experiment

### Test equipment

**Figure 8:**
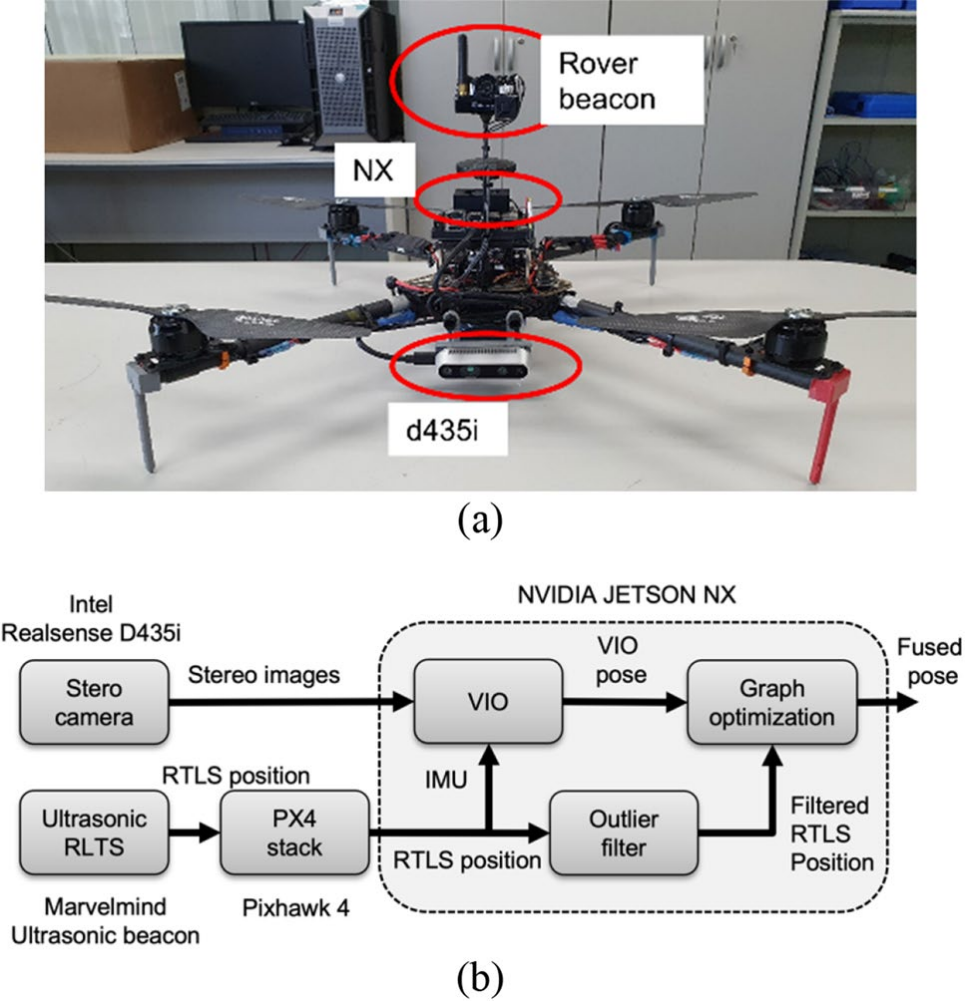
A drone for the experiment (**a**) and its hardware and software architecture (**b**).

A drone for evaluation of localization performance is shown in Fig. 8a and its onboard computers and sensors are listed in Table 3. Figure 8b illustrates the hardware and software architecture of the test drone. The Marvelmind ultrasonic beacon sends RTLS position data in the form of NMEA (National Marine Electronics Association) GPS messages to the PX4 Firmware stack in Pixhawk 4 FCC via UART. This communication occurs at a rate of 8–9 Hz. The Intel Realsense D435i provides stereo images to the NVIDIA Jetson NX at a rate of 30 Hz. The Pixhawk 4 transmits IMU and RTLS position data to the NVIDIA Jetson NX via the Robot Operating System (ROS) platform. On the NVIDIA Jetson NX, the VIO, RTLS outlier filter, and graph optimization algorithm are executed.

**Table 3:** Onboard computers and sensors.

Component	Model
Flight control computer	Pixhawk 4
Companion computer	Jetson Xavier NX
Camera for VO	Intel Realsense D435i
Ultrasonic rover beacon	Marvelmind ultrasonic beacon
Real-time kinematic (RTK) GPS	Here3 RTK GPS

### Hangar experiment

Due to safety restrictions on flying inside a hangar, rather than conducting flight tests, we performed two manual tests in a hangar environment equipped with ultrasonic beacon RTLS.

First, a test drone suspended beneath a hoist moved around a Boeing B737-800 as shown in Fig. 1a in path B of Fig. 6a. The path B measures 6.0 by 9.0 m, forming a rectangular shape, and the total distance covered by the drone during the test was 36.12 m. In this test case, the capability of localization continuity even in a situation of unstable ultrasonic RTLS is tested for the suggested fusion localization system.

Second, a tester moved manually with a drone following a 16.2 X 7.2 m rectangular path, covering a total distance of 46.8 m. (path A of Fig. 6a). Reference positions in path A were acquired and time-synchronized with the estimated position data, allowing for relative comparison, as explained in "Visual odometry algorithm" section. This test aims to evaluate whether fusion localization with RTLS improves accuracy better than VIO-only localization in terms of RMSE (Eq. 8) and final translation drift relative to trajectory length (TDr) (Eq. 9)^21^.

8 $$RMSE=\sqrt{\frac{1}{n}{\sum }_{i=1}^{n}\Vert \widehat{{p}_{i}}-{p}_{i}\Vert }$$

where $\widehat{{p}_{i}}$ is estimated position and ${p}_{i}$ is reference position.

9 $$TDr=\frac{\Vert {\widehat{p}}_{final}-{p}_{final}\Vert }{L}$$

where ${\widehat{p}}_{final}$, ${p}_{final}$, and L are the estimated final position, the reference final position, and total length, respectively. In this rectangular A path test, the reference final position of the trajectory coincides with the reference start point.

### Flight experiment

For the flight test, an ultrasonic beacon system was installed in an open outdoor site, which has similar characteristics to a hangar floor in terms of insufficient feature points and far distance to any detectable objects as shown in Fig. 11. In this outdoor environment, we tested the automated flight capability of the suggested fusion localization system by conducting guided-mode flight following predetermined waypoints.

## Result

### Hangar test #1—stable ultrasonic RTLS

**Figure 9:**
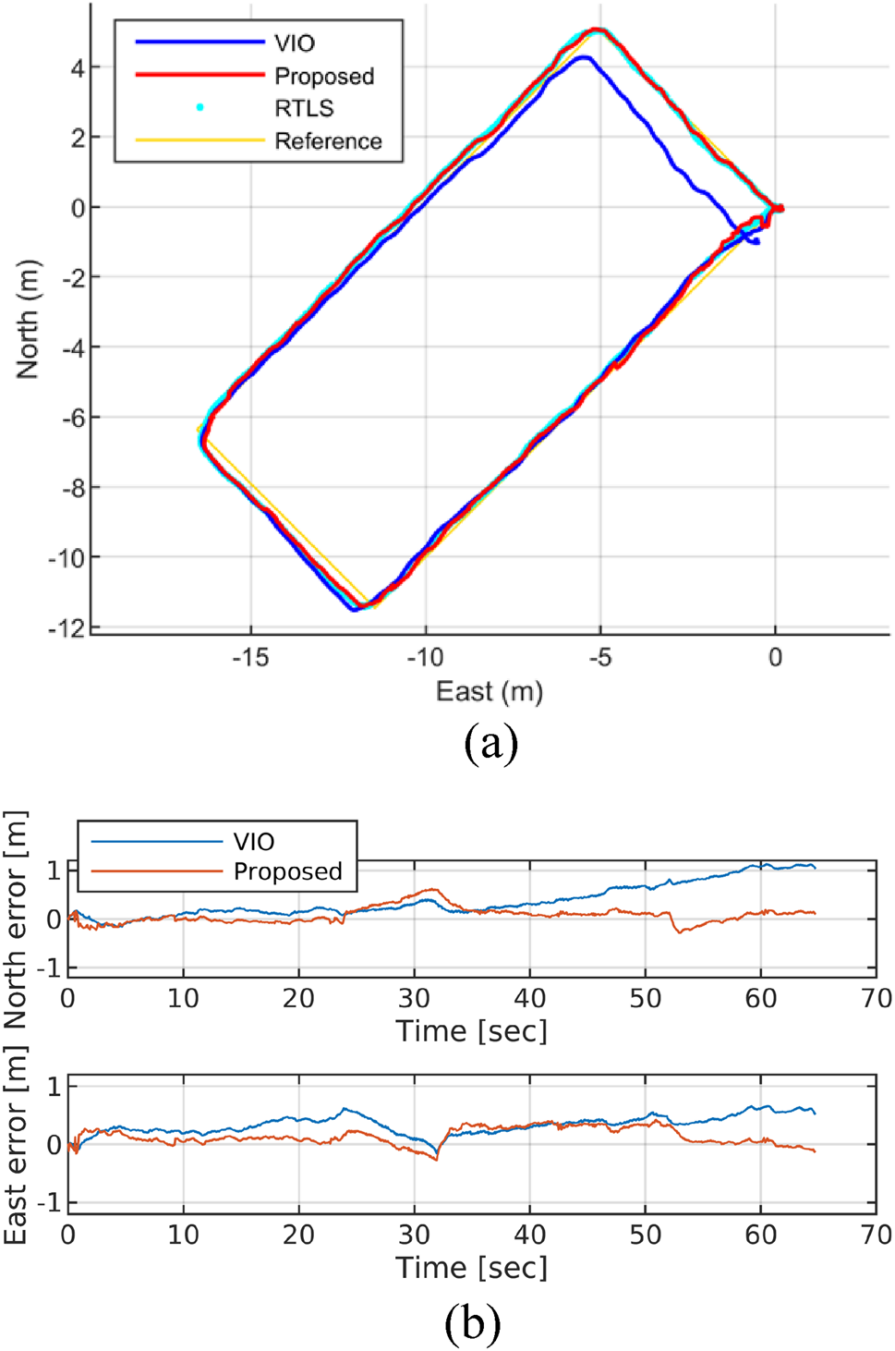
Proposed algorithm and VIO test result: RTLS improves localization accuracy.

The test result of the stable ultrasonic RTLS case, where the drone manually moved along path A as shown in Fig. 6a, is presented in Fig. 9. While the VIO-generated trajectory exhibits significant error accumulation upon returning to the starting position at (0, 0), the suggested fusion solution effectively compensates for this error using RTLS data, resulting in smaller RMSE and TDr values (Fig. 9 and Table 4).

**Table 4:** Hangar test result.

	VIO	Proposed
RMSE (m)	0.5995	**0.2880**
TDr (%)	2.45	**0.36**

Significant values are in bold.

### Hangar test #2—unstable ultrasonic RTLS

**Figure 10:**
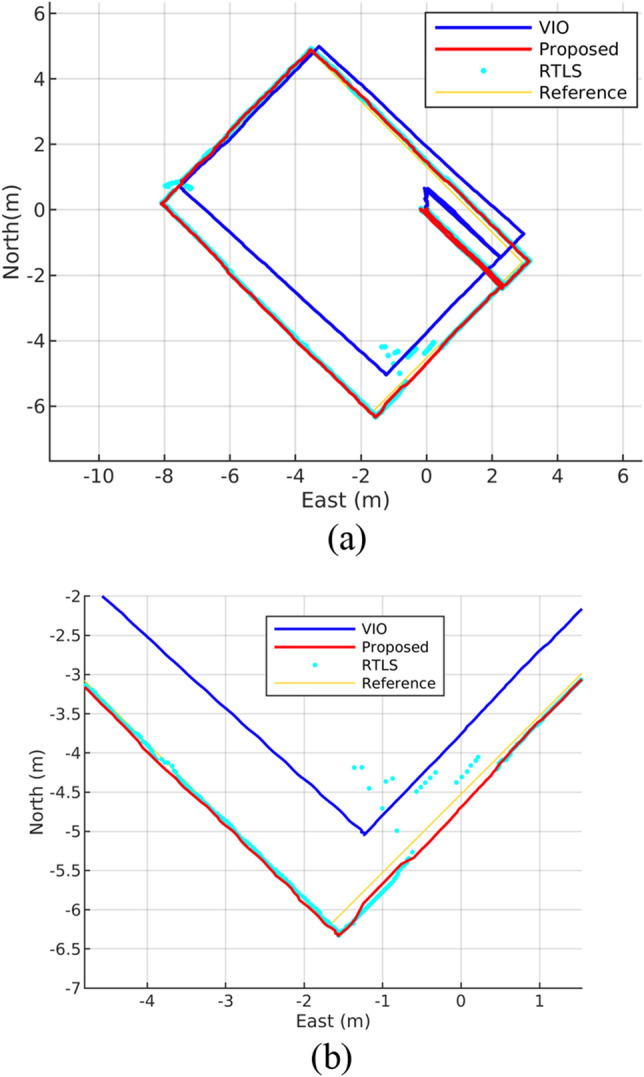
Proposed algorithm and VIO test result (**a**) and RTLS unstable region view (**b**): RTLS fails but localization continues.

The test result of the unstable ultrasonic RTLS case, in which the drone hanging from the hoist moved along path B as shown Fig. 6a, is presented in Fig. 10. At coordinates of (0, − 5) and (− 8, 1) where RTLS data are unstable, it can be seen that localization of the proposed algorithm is not affected by unstable RTLS data (Fig. 10b) thanks to outlier filter which prevents RTLS data from being inserted in the optimization graph (Fig. 6c). In addition, the calculated trajectory shows that VIO experiences significant horizontal error at coordinate (0, 0) during lift-off from the hangar floor. This error is primarily attributed to the restricted camera FOV and the lack of sufficient feature points on the floor. In contrast, our proposed algorithm adjusts the error through fusion with ultrasonic RTLS.

### Flight test

**Figure 11:**
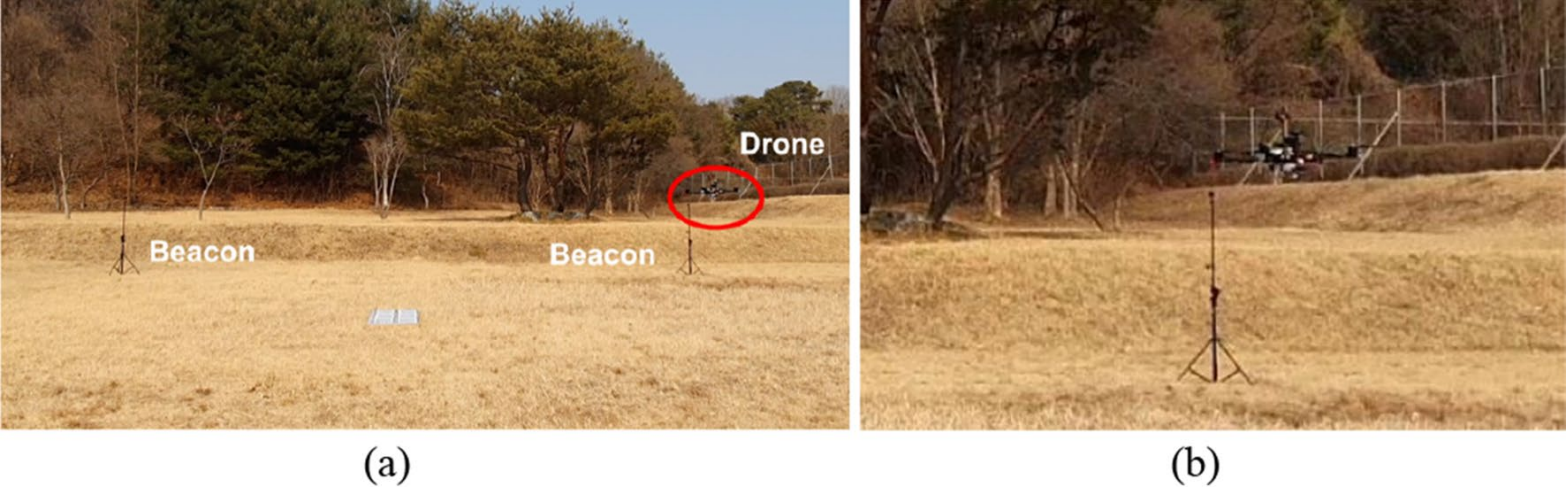
Outdoor flight test (**a**) and zoomed-in image (**b**).

**Figure 12:**
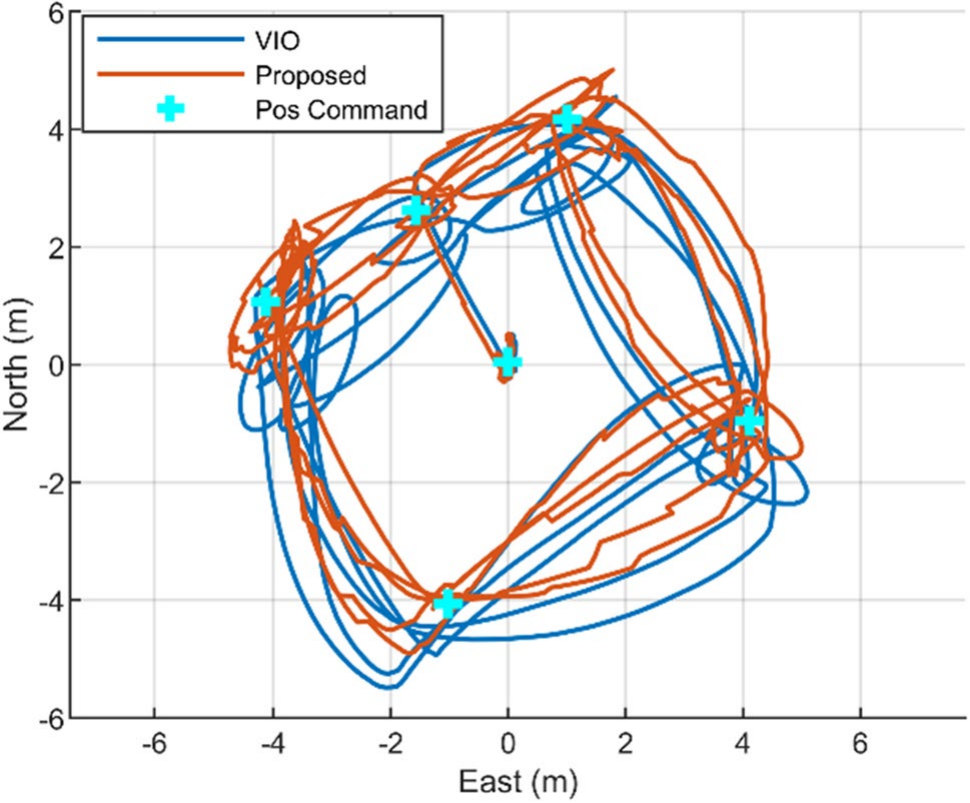
Outdoor flight test trajectory (VIO position output, proposed algorithm position output, and commanded position (waypoints)).

The outdoor flight test result shows that the suggested fusion localization system can fly a drone in a guided mode (Fig. 11). Waypoints were set to create a path turning around 6 X 6 square repeatedly, and the drone flew automatically, following theses waypoints. The total flight length covered was 189 m. The test result affirms that suggested localization system is capable of real-time navigational performance (Fig. 12). Through the real-time implementation, the position estimation period of the suggested algorithm in the target board is 14.35 Hz based on feature inserting period of 15 Hz, which is 95.6%. Thanks to images and feature marginalization and usage of the GPU, calculation delay is avoided and flight can be conducted successfully. Additionally, Table 5 shows that the localization result of the proposed algorithm is more accurate than VIO.

**Table 5:** Outdoor flight test result.

	VIO	Proposed
RMSE (m)	0.8388	**0.5778**
TDr (%)	0.67	**0.14**

Significant values are in bold.

## Conclusion

In this paper, a fusion localization system of VIO and ultrasonic RTLS for airplane drone inspection is proposed. In configuring the VIO system, we determined that an optical flow algorithm utilizing stereo images with view direction turned away from an airplane is the most suitable choice for a hangar environment. To utilize ultrasonic RTLS which has inherent drawbacks in non-LOS situations, an outlier filter is suggested. Then, VIO and outlier-filtered ultrasonic RTLS localization are fused through graph optimization. Experiment results in a hangar demonstrate that the proposed system effectively compensates for accumulated drift error in VIO in case of stable RTLS conditions. Also, the test result shows that in case of unstable RTLS conditions, abnormal RTLS data is rejected and localization can continue using VIO without RTLS. We implemented image buffer and graph marginalization in VIO and graph optimization for real-time capability on the NVIDIA Xavier NX target board. Using these methods, we successfully accomplished an automated drone flight.

As future research for more stable fusion localization, we plan to develop a system that can change into RTLS fused with IMU mode in situations of VIO divergence or tracking loss. Furthermore, we are considering the use of a more powerful single-board computer to enhance calculation speed and accuracy.

## Data Availability

The datasets generated and analyzed during the current study are not publicly available due to aviation security in a hangar facility but are available from the corresponding author upon reasonable request.
